# Host–Virus Immune Interactions in Andes Virus Infection: Implications for Human-to-Human Transmission, Pathogenesis, and Public Health

**DOI:** 10.3390/cimb48101050

**Published:** 2026-10-12

**Authors:** Awadh Alanazi, Mohamed N. Ibrahim, Maram Awied Alenezi, Rahaf Ziyad I. Alabdullah

**Affiliations:** 1 Department of Clinical Laboratory Sciences, College of Applied Medical Sciences, Jouf University, Sakaka 72388, Saudi Arabia; 2 Department of Clinical Laboratories Sciences, College of Applied Medical Sciences at Al Qurayyat, Jouf University, Al Qurayyat 77454, Saudi Arabia; 3 Northern Borders Health Cluster, Almosaediah, Arar 73311, Saudi Arabia; 4 Medical Internship, College of Medicine, Jouf University, Sakaka 72388, Saudi Arabia

**Keywords:** hantavirus cardiopulmonary syndrome, vascular leakage, interferon signaling, immune evasion, cytokine dysregulation, viral kinetics, infectiousness, contact tracing, genomic epidemiology

## Abstract

The Andes virus (ANDV, *Orthohantavirus*, andesense) is unique among hantaviruses in that it may transfer from person to person and cause severe vascular damage. The evidence on host–virus immunological interactions and their importance in disease progression, transmission, and public health is compiled in this narrative review. ANDV mostly affects pulmonary endothelial cells and does not result in widespread cell loss. Rather, the loss of vascular barrier integrity is caused by vascular endothelial growth factor (VEGF) signaling, Ras homolog family member A (RhoA) activation, breakdown of vascular endothelial (VE)-cadherin, hypoxia-related pathways, and mediators produced from infected pericytes. Additionally, the virus interferes with early antiviral defenses involving protein kinase R (PKR), TANK-binding kinase 1 (TBK1)–interferon regulatory factor 3 (IRF3), mitochondrial antiviral-signaling protein (MAVS), and retinoic acid-inducible gene I (RIG-I)/melanoma differentiation-associated protein 5 (MDA5). The intensity or outcome of the disease has been associated with increased interleukin-6 (IL-6), altered complement activity, thrombocytopenia, neutralizing-antibody reactions, and specific host genetic variations; nevertheless, significant T-cell activation does not necessarily correspond with viral clearance. Blood and a number of mucosal and secretory samples have been found to contain ANDV ribonucleic acid (RNA), albeit RNA discovery by itself does not prove the presence of an infectious virus. Epidemiological evidence indicates that Secondary transmission is rare and mostly linked to extended close or intimate contact. Genomic sequencing, when paired with epidemiological evidence, can shed light on transmission chains. Recent outbreaks linked to travel further emphasize the significance of worldwide surveillance and coordinated contact tracing. Prospective studies integrating genomic analysis, serial virological sampling, and standardized exposure evaluation are required to more effectively define the infectious phase and transmission patterns

## 1. Introduction

Rodent-borne orthohantaviruses linked to human illness are tri-segmented, negative-sense RNA viruses categorized within the family Hantaviridae, subfamily Mammantavirinae, and genus *Orthohantavirus*. The Andes virus (ANDV; *Orthohantavirus andesense*), which is the primary cause of hantavirus cardiopulmonary syndrome (HCPS) in southern South America, continues to be one of the most clinically significant New World hantaviruses due to the fact that reported fatality rates in severe HCPS can reach 50% [1]. The sudden transition from a vague febrile illness to severe respiratory compromise accompanied by widespread capillary leakage, pulmonary edema, and circulatory instability is the hallmark of ANDV-induced HCPS. Experimental evidence showed that human primary lung endothelial cells infected with the ANDV had decreased VE-cadherin expression along with increased permeability and loss of the integrity of the endothelial cell barrier [1]. Crucially, this vascular dysfunction happens without significant endothelial cytolysis, suggesting that severe illness is more likely to be caused by changes in host–virus signaling and barrier modulation than by direct pulmonary endothelium damage.

ANDV is mostly found in Argentina and Chile, where its ecology is strongly associated with reservoirs of sigmodontine rodents and environmental conditions such as habitat type, temperature, precipitation, and humidity [2]. In endemic settings, human infection is typically linked to forestry activities, agricultural occupations, or rural exposure [3]. As previously reported, ANDV circulation in Chile was linked to different ecogeographic areas and disparate landscapes with significant variations in flora, temperature, precipitation, and humidity [4]. A study in northwest Patagonia reported that peridomestic habitat change was associated with greater opportunities for human exposure to ANDV and favored *Oligoryzomys longicaudatus* abundance [5]. A strong correlation was reported between hantavirus disease, precipitation and habitat type characteristics in Latin America and the Caribbean with rainfall showing the strongest positive link with human hantavirus infections [5]. These results suggest that human exposure, environmental factors, and reservoir ecology interact to influence ANDV emergence instead of reservoir presence only.

Even though human ANDV infection is typically contracted through contact with infected rodents, ANDV is the only hantavirus for which limited person-to-person transmission has been reported [6]. This route of transmission is still rare, though, and it has not resulted in long-term community spread. Evidence currently available indicates that secondary transmission is rare and mostly linked to extended intimate contact with symptomatic patients, especially in the early stages of illness, as opposed to casual contact or persistent population-level transmission [6,7]. Even though ANDV infection can cause delayed viral clearance and high immune activation [8], it is still unknown why some infected individuals spread the virus while others do not. The roles of viral burden, host response, and exposure time in this distinction are still unclear and no host immunological signature has been demonstrated to differentiate transmitters from non-transmitters. Thus, recent epidemiological, immunological, and molecular research supports the separation of two related but distinct problems. Endothelial barrier failure and clinical severity are increasingly explained by host–virus interactions, although the factors influencing individual infectiousness and transmission heterogeneity are still largely unknown. Although viral RNA has been found in blood and a number of mucosal and secretory compartments, the existence of an infectious virus or the capacity for transmission cannot be determined solely by RNA positivity. Similarly, genetic similarity improves the inference between cases that are epidemiologically connected, but it cannot independently determine the direction of transmission or rule out shared exposure. Concern has now spread beyond historically endemic rural areas due of the potential for exposure to spread across several jurisdictions through international travel. A multinational response to the 2026 MV Hondius outbreak included medical evacuation, repatriation, coordinated international contact tracing, isolation, quarantine, and clinical and laboratory monitoring of those who were exposed. The outbreak shows that when potentially exposed persons spread abroad, even a little amount of ANDV transmission can result in significant public health needs [9].

ANDV transmission and cardiopulmonary disease, including viral shedding, clinical characteristics, and treatment, have been reviewed recently. This narrative review focuses on whether the timing or length of infectiousness and secondary transmission may be influenced by host immune responses linked to severe illness or viral control. It combines data on viral shedding, clinical immunological findings, mechanistic research, and epidemiological proof of transmission.

### Methods

Combinations of “Andes virus,” “hantavirus cardiopulmonary syndrome,” “immune response,” “viral shedding,” “person-to-person transmission,” and “outbreak” were used to search PubMed, Scopus, and Web of Science from inception to June 2026. Included were English-language reviews and research pertinent to ANDV pathophysiology, host immunity, viral persistence or shedding, transmission, and outbreak response. We looked through the reference lists of pertinent publications to find more relevant reports. Animal studies were taken into account for mechanistic evidence while human research and epidemic investigations were used to evaluate clinical, epidemiological, and transmission data, as well as cell-culture information.

## 2. Host–Virus Immune Interactions

### 2.1. Viral Entry and Endothelial Tropism

**Figure 1:**
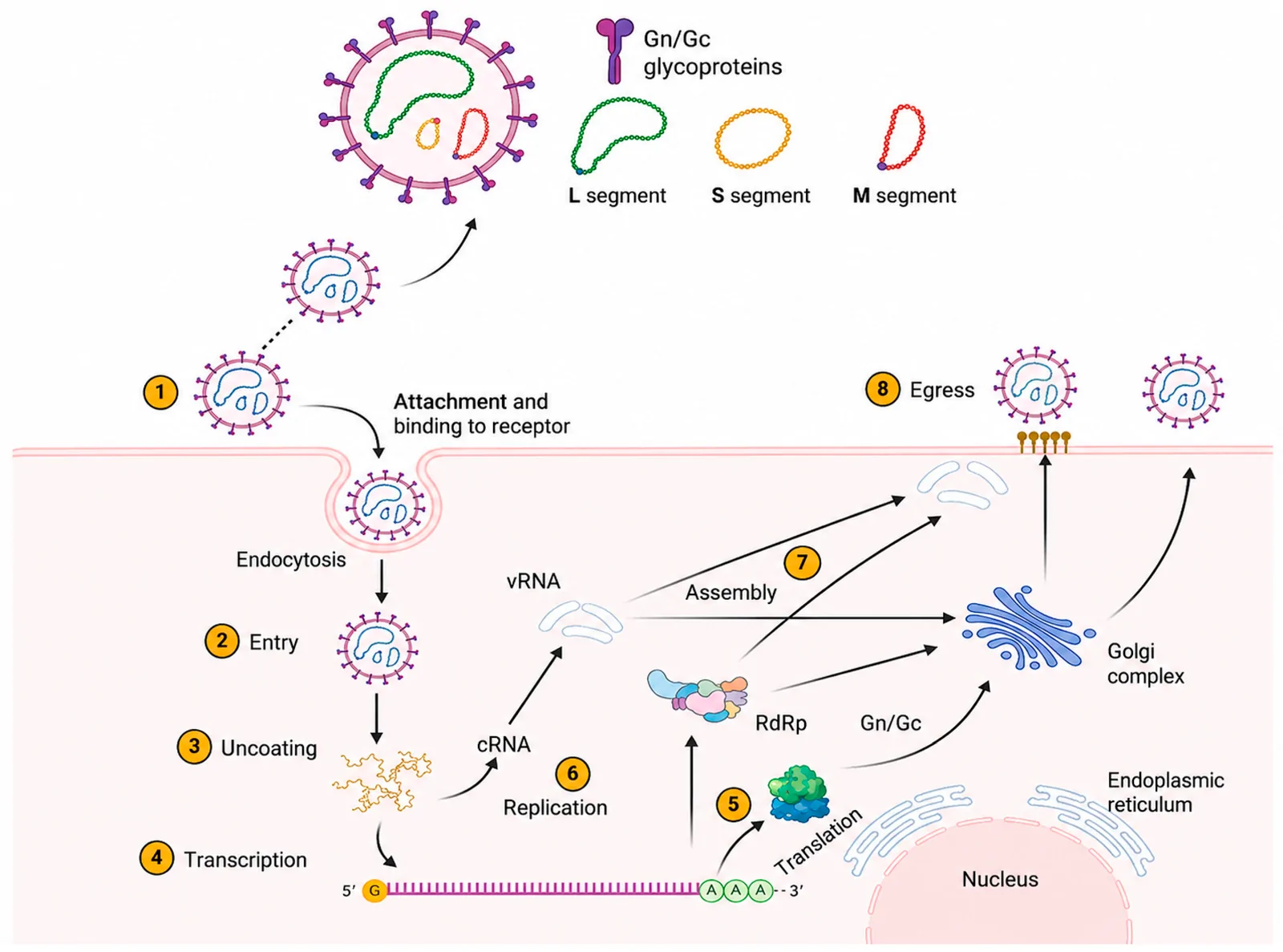
Structure and life cycle of hantaviruses. Three negative-sense RNA genome segments (S, M, and L) are present in the virion’s envelope, which is made up of Gn/Gc glycoproteins. (1) Attachment: host–cell receptors, such as β3 integrin and protocadherin-1 (PCDH1), are engaged by viral glycoproteins. (2) Entry: ANDV is internalized via endocytic routes, mostly dependent on clathrin, dynamin, and cholesterol; a clathrin-independent mechanism has also been documented. (3) Viral ribonucleoprotein complexes are discharged into the cytoplasm during uncoating. (4) Transcription: The viral RNA-dependent RNA polymerase transcribes viral messenger RNA from the genome using capped primers obtained from the host. (5) Translation: Viral messenger RNAs are transformed into viral proteins by host–cell machinery. (6) Replication: complementary RNA is created by the viral polymerase and used as a template to create fresh viral genomic RNA. (7) Assembly: To create offspring particles, viral genomic ribonucleoproteins bind to viral glycoproteins. (8) Egress: newly created particles depart from the diseased cell. Created with BioRender.com (2026). https://BioRender.com/3e4kylq (accessed on 27 July 2026).

Attachment and receptor engagement are the first steps in the usual hantavirus replication cycle, which is followed by endocytic uptake, uncoating, transcription and replication, and virion assembly and release (Figure 1). Receptor recognition and cell entry are two of these processes that are essential to comprehending endothelial tropism. Vitronectin, antibodies against αvβ3 integrins, and β3-integrin PSI-domain polypeptides produced from humans and Syrian hamsters suppressed ANDV infection in tests employing human umbilical vein endothelial cells (HUVECs) and Syrian baby hamster kidney (BHK-21) cell. Significantly, this inhibitory effect was removed when the L33P mutation was added to the human β3 PSI domain, indicating that the β3-integrin PSI domain plays a residue-specific role in ANDV–host cell contact [10]. Protocadherin-1 (PCDH1) was found to be a crucial factor in ANDV and Sin Nombre virus entry and infection of pulmonary endothelial cells in later research. PCDH1 is directly recognized by their surface glycoproteins, and Syrian golden hamsters lacking PCDH1 exhibited strong resistance to an ANDV assault that is often fatal [11]. A small interfering RNA (siRNA) screen of 140 membrane-trafficking genes in primary human lung microvascular endothelial cells (HMVEC-L) revealed that ANDV entry was mostly dependent on clathrin, dynamin, and membrane cholesterol. The findings also showed a second clathrin-independent entry pathway with characteristics of micropinocytosis [12].

Overt cell lysis is not primarily linked to ANDV-induced endothelial infection. Instead, experimental research indicates that the virus disrupts signaling pathways that typically maintain the integrity of the endothelium barrier, increasing vascular permeability. Replicating ANDV enhanced monolayer permeability, reduced VE-cadherin levels, and raised VEGF production in primary human pulmonary microvascular endothelial cells from individual donors. A direct connection between ANDV replication and VEGF–VEGFR2-mediated breakdown of adherens junctions is supported by the considerable restoration of VE-cadherin upon inhibition of VEGFR2 [1]. RhoA signaling was found to be another factor in barrier dysfunction in a different investigation. In primary pulmonary microvascular endothelial cells, ANDV infection or production of the viral nucleocapsid protein alone triggered RhoA. ANDV-associated permeability was reduced by roughly 80–90% when RhoA activity was reduced by knockdown, dominant-negative RhoA, or the Ras homolog family member A/ Rho-associated coiled-coil-containing protein kinase (RhoA/ROCK) inhibitors fasudil and Y27632 [13]. This suggests that RhoA activation independently contributes to endothelial barrier disruption.

In primary human pulmonary micro-vascular endothelial cells, the molecular underpinnings of this RhoA response were later identified. Instead of directly binding RhoA, the ANDV nucleocapsid protein bound and sequestered its inhibitor, Rho GDP-dissociation inhibitor (RhoGDI), which decreased the amount of RhoGDI available to keep RhoA in an inactive state. Additionally, nucleocapsid protein increased the release of RhoA from S34-phosphorylated RhoGDI under hypoxia- or VEGF-driven protein kinase C alpha (PKCα) signaling, increasing RhoA activation, VE-cadherin internalization, and endothelial permeability [14]. Therefore, ANDV can compromise ad-herens-junction stability without endothelial cell death through complimentary pathways such as VEGF–VEGFR2 signaling and nucleocapsid-mediated RhoGDI–RhoA dysregulation.

Severe HPS-related hypoxic circumstances may intensify these endothelial reactions even more. Hypoxic exposure of ANDV-infected monolayers in primary human pulmonary microvascular endothelial cells and pulmonary lymphatic endothelial cells raised permeability three to six times, enhanced transcription of hypoxia-inducible factor 1 (HIF1)-responsive VEGF-A, angiopoietin-4, and EGLN3, and elevated ribosomal protein (S6 kinase) phosphorylation. Rapamycin supported the role of the mechanistic target of rapamycin (mTOR)–S6K pathway in hypoxia-enhanced endothelial dysfunction by inhibiting mTOR signaling and preventing the permeability response and giant-cell formation [15].

Perivascular cells may also be impacted by ANDV’s vascular actions. Primary human vascular pericytes were infected for up to nine days, and conditioned media from infected pericytes both boosted VEGF secretion and the permeability of pulmonary microvascular endothelial monolayers [16]. Together with the endothelial studies, these results demonstrate that ANDV can impair barrier function through reactions involving both endothelial cells and pericytes. Since most of the information comes from in vitro research, their respective contributions during human HCPS are still unknown [1,13,14,15,16].

### 2.2. Innate Immune Evasion

Evidence now available indicates that ANDV postpones the early innate antiviral response rather than totally suppressing it. Compared to the nonpathogenic Prospect Hill virus, ANDV significantly reduced the early generation of interferon beta (IFN-β), Interferon Regulatory Factor 3 (IRF3) dimerization, and IRF3 nuclear translocation in primary human pulmonary microvascular endothelial cells from individual donors. Both viruses decreased IFN-β-induced signal transducer and activator of transcription 1/2 (STAT1/STAT2) activation in complementary tests in Vero E6 cells, indicating that their primary distinction is at the level of early IRF3-dependent interferon induction rather than through an ANDV-specific inhibitor of downstream IFN signaling [17]. In human umbilical vein endothelial cells, ANDV replication also induced myxovirus resistance protein 1 (MxA) RNA and protein, but the MxA response was less pronounced in A549 cells. Lower ANDV permissiveness was linked to higher MxA induction, and constitutive MxA expression in Vero E6 cells significantly decreased viral replication [18].

Viral factors have been discovered through mechanistic studies as potential contributors to this early signaling dysfunction. The ANDV nucleocapsid protein suppressed signaling via retinoic acid-inducible gene I (RIG-I), melanoma differentiation-associated protein 5 (MDA5), mitochondrial antiviral-signaling protein (MAVS), TANK-binding kinase 1 (TBK1), and IκB kinase epsilon (IKKε) in human embryonic kidney 293T (HEK293T) reporter assays by blocking TBK1 autophosphorylation and the subsequent phosphorylation of IRF3. Prospect Hill, New York-1, and Sin Nombre virus nucleocapsid proteins did not exhibit comparable inhibition [19]. Additional mutational research revealed that the ANDV nucleocapsid protein’s inhibitory effect on IRF3 phosphorylation and interferon induction was eliminated when Ser386 was substituted with histidine or alanine; however, this activity was restored by the phosphomimetic S386D substitution. Phosphorylation at Ser386 in the nucleocapsid protein from ANDV-infected cells was also shown by mass spectrometry [20].

For the ANDV NSs protein, a different mechanism has been discovered. NSs expression was found in lung tissue from experimentally infected male golden Syrian hamsters while research in virus-free HEK293T and Huh-7 cells revealed that NSs interacted with MAVS and decreased MAVS-dependent IFN-β, NF-κB, IRF3, and ISRE signaling without interfering with the interaction between MAVS and TBK1 [8]. Additionally, a subsequent interferon-effector pathway was impacted by the ANDV nucleocapsid protein. Studies in HUVEC, Huh7, HEK293T, and Vero E6 models demonstrated that ANDV N inhibited PKR dimerization and autophosphorylation, thereby decreasing PKR-dependent translational restriction despite enhanced PKR expression during infection [21].

Animal research also supports the significance of early innate immune timing. SNV infection administered one day prior to ANDV challenge avoided fatal illness in Syrian hamsters, and pretreatment with the innate immune stimulation poly I:C offered comparable protection. Conversely, the mice were not protected when SNV and ANDV were administered at the same time [22]. Whole-blood NanoString research revealed that RIG-I-like receptor and interferon pathway activation happened later during ANDV infection in a different hamster investigation comparing deadly ANDV infection with asymptomatic Hantaan virus infection. Broader transcriptional alterations emerged as viremia rose with complement, coagulation, and apoptosis-related pathways becoming more prominent at later stages [23].

When combined, cell and animal research points to a temporal paradigm where ANDV does not totally inhibit the response but rather delays early innate antiviral signaling through many viral elements. The existing data does not show the relative contributions of these processes to human disease [8,16,17,18,19,20,21,22].

### 2.3. Inflammatory Dysregulation

Clinical research reveals that inflammatory reactions during ANDV infection are diverse, with some mediators exhibiting more robust correlations with the severity of the illness than widespread cytokine increase. At hospital admission, serum levels of tumor necrosis factor alpha (TNF-α), interferon gamma (IFN-γ), interleukin 10 (IL-10), and interleukin 6 (IL-6) were considerably higher in 43 Chilean patients with proven ANDV infection compared to 43 healthy controls. There were 21 mild and 22 severe instances in the patient cohort; 14 of them later passed away. While TNF-α, IFN-γ, and IL-10 did not significantly change based on severity, IL-6 concentrations were almost 40 times greater in severe than moderate instances [24]. This differential correlation shows that IL-6 is more closely linked to clinical development and refutes the interpretation of severe HCPS as uniform cytokine amplification.

A wider systemic inflammatory profile was shown in a larger retrospective cohort of 93 Argentine patients with confirmed ANDV-associated HPS, including 34 fatal cases. Serum levels of IFN-γ, soluble TRAIL, intestinal fatty acid-binding protein, IL-6, and IL-10 were higher in fatal cases than in survivors. Intestinal fatty acid-binding protein was found to be independently linked with a fatal result and IL-6 with the severity of the disease, according to multivariable analysis [25]. Although these indicators by themselves do not prove systemic vascular leakage, elevated markers of intestinal epithelial damage and microbial translocation further suggest that severe illness involves systemic mechanisms beyond the pulmonary inflammatory response [25].

Immune activation may affect endothelial barrier function, according to experimental findings. After being infected with the clinical ANDV strain CHI-7913, human immature dendritic cells isolated from peripheral blood monocytes developed a largely mature pro-inflammatory phenotype with elevated TNF-α and active MMP-9. TGF-β decreased more significantly, but IL-10 altered just slightly [26]. The permeability of primary HUVEC monolayers was greatly increased by supernatants from infected dendritic cells, indicating that soluble products released from ANDV-infected immune cells can modify endothelial barrier function without directly infecting the endothelial cells in that experiment [26].

**Figure 2:**
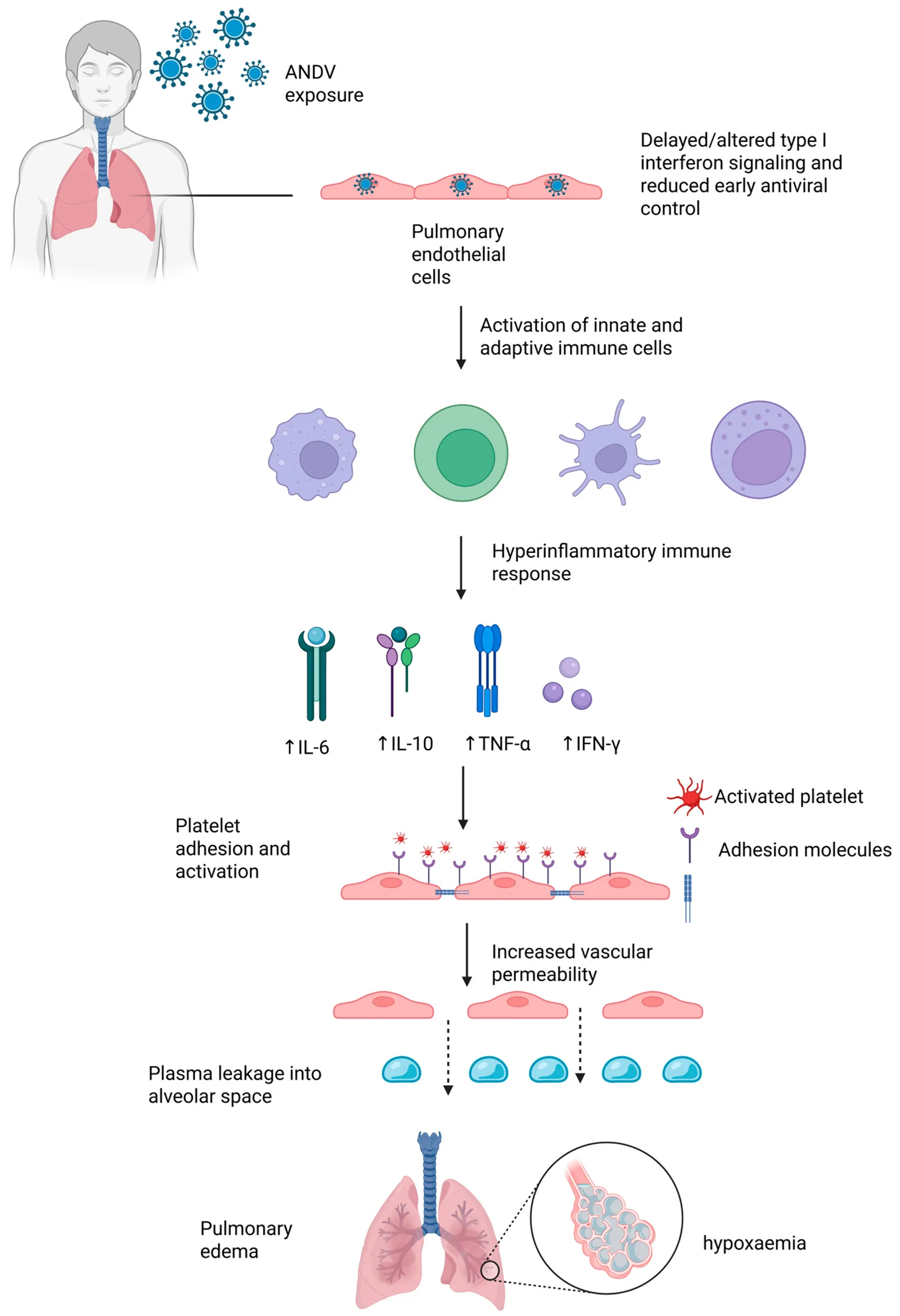
Immune dysregulation during Andes virus infection. Solid arrows indicate the schematic progression of immune activation, inflammatory responses, and increased vascular permeability. Dashed arrows represent fluid leakage across the endothelial barrier into the alveolar space, while dotted connecting lines indicate the magnified pulmonary region. Created in BioRender. S. (2026) https://BioRender.com/agw6iol (accessed on 27 July 2026).

The sequence shown in Figure 2 incorporates results from independent investigations on vascular leakage, platelet and endothelial responses, immune-cell activation, delayed or altered type I interferon signaling, and cytokine alterations. These results collectively indicate a suggested development toward plasma leakage, pulmonary edema, and hypoxia, although no single study has shown the entire process.

### 2.4. Adaptive Immunity and Viral Control

Studies on humans show that there is significant adaptive immune activation during ANDV infection, even though the amount of lymphocyte activation by itself does not always correlate with viral clearance. ANDV RNA was found in peripheral blood cells up until day 53 following hospital admission in a longitudinal case study of a 56-year-old Chilean survivor with severe HCPS; by day 88, it was no longer detectable. Increased granzyme B secretion and a significant rise in Gn46–60-specific IFN-γ-producing CD8+ T cells were associated with the loss of detectable viral RNA, whereas neutralizing-antibody titers stayed relatively stable. However, the authors acknowledged that it was impossible to prove that these T cells produced viral clearance using the single-patient approach [27].

Additionally, 78 Chilean patients tested between four months and 13.2 years following ANDV infection had their long-term cellular immunity assessed. The majority of the detectable T-cell response was attributed to Gn-derived epitopes, although Gn-specific CD8+ T cells continued to be detectable for over 13 years and were still able to produce TNF-α, granzyme B, and IFN-γ. Ten of the 17 people who were monitored throughout time had higher neutralizing-antibody titers. The study did not, however, ascertain whether these enduring antibody or cellular responses were required for defense against reinfection [28].

A straightforward link between the quantity of activated T cells and effective virus suppression is undermined by subsequent clinical data. Immunological analyses revealed high frequencies of activated CD4+ and CD8+ T lymphocytes during acute disease in a cohort of 154 Argentine HPS patients tested between 2014 and 2019, with no discernible variations between moderate and severe cases. However, viral RNA was still detectable while the patient was in the hospital and in certain cases while they were recovering. Longitudinal assessments revealed that clearance typically occurred as neutralizing-antibody titers increased, although one patient remained RNA-positive 202 days after fever onset despite high neutralizing titers [29]. These results imply that response kinetics and antigen-specific function may offer more information than the overall number of activated T cells when compared to the previous single-patient study.

Additionally, humoral immunity varies. Circulating plasmablast counts rose significantly and peaked about a week after symptom onset in 17 Argentine patients with acute ANDV-associated HPS. Plasmablast quantity was not linked to the severity of the sickness or viraemia, and reactivity against both viral and unrelated antigens revealed that the response involved the generation of virus-specific antibodies in addition to significant polyclonal B-cell activation [30].

Experimental intervention trials provide more proof of antibody-mediated protection. 29 patients with proven HCPS were given convalescent immune plasma with ANDV-neutralizing antibodies in a non-randomized multicenter Chilean study. A definitive efficacy result was not possible due to the non-randomized design and borderline statistical significance; however, mortality was 14% among treated patients compared with 32% among contemporaneous untreated cases nationally [31]. Therefore, this study offers suggestive clinical evidence for antibody-based treatment rather than direct evidence that ANDV disease severity is predicted by admission neutralizing-antibody titers.

Screening sera from 27 ANDV convalescent patients and extracting glycoprotein-specific memory B cells from a highly neutralizing donor provided more direct experimental data. When given to Syrian hamsters following infection, human monoclonal antibodies (mAbs), JL16 and MIB22, effectively neutralized ANDV and each offered total protection against a deadly ANDV challenge [32]. One ANDV survivor produced 16 ANDV-reactive monoclonal antibodies, while three SNV survivors produced 20 antibodies. While ANDV-44 had more widespread neutralizing action, the majority of ANDV-derived antibodies were species-specific, and four particular antibodies offered post-exposure protection in the fatal hamster model at relatively low doses [33].

More recent human data indicates that antibody activities other than neutralization may be involved in protective humoral immunity. Higher Gn-specific IgG levels, as well as increased antibody-dependent natural killer (NK)-cell activation and complement deposition, were linked to moderate disease among 14 patients with acute HCPS, comprising six moderate and eight severe cases. Years after infection, Fc receptor-activating and other polyfunctional antibody functions were still detectable in a different examination of 34 survivors [34].

Human studies link certain adaptive immune responses to virus clearance or illness prognosis while antibody treatments have shielded hamsters from a deadly ANDV challenge. Nevertheless, present clinical data does not demonstrate the effectiveness of antibody treatment in patients or establish a validated immunological marker that predicts viral clearance or disease severity [26,27,28,29,30,31,32,33,34].

### 2.5. Immune Correlates of Disease Severity

Clinical research indicates that certain inflammatory, hematological, and host-genetic characteristics are linked to ANDV severity instead of the total level of immune activation. Since Section 2.3 discusses the inflammatory profile, the results presented here are mainly evaluated in terms of their correlation with clinical outcome. Compared to TNF-α, IFN-γ, or IL-10, IL-6 was more strongly associated with severe disease, but intestinal fatty acid-binding protein and complement C5/C5a were clearly associated with survival and fatal prognosis, respectively [24,25]. These results imply that specific elements of the inflammatory response may be more predictive than widespread cytokine increase.

A more easily accessible clinical correlation is platelet count. Of the 175 patients with proven ANDV infection in a retrospective Chilean cohort, 40 had mild illness at first, while 14 later had moderate or severe HCPS. Patients who advanced had median platelet counts of 37 K/mm^3^ at admission, while those who stayed mild had median platelet counts of 83 K/mm^3^. In this cohort, a platelet count more than 115 K/mm^3^ strongly distinguished non-progressive illness; nevertheless, independent validation is necessary before this threshold can be regarded as widely predictive [35].

Additionally, host-genetic research indicates that each infected person has a different vulnerability to severe illness. Human leukocyte antigen (HLA)-DRB115 was significantly more common in moderate disease in 87 Chilean individuals, while HLA-B08 was more common in severe HCPS, however the latter correlation was only marginally significant [36]. IL28B polymorphisms rs12979860 and rs8099917 were linked to clinical severity in a larger analysis of 238 verified cases, but TNF-α rs1800629 did not demonstrate any correlation with outcome [37].

Additional proof of host-genetic variability comes from copy-number variation. Signal-regulatory protein beta 1 gene (*SIRPB1*) deletion was more common in moderate disease among 195 Chilean patients, while complement factor H-related 1 and 3 genes (*CFHR1*/*CFHR3*) deletion was more common in severe HCPS [38]. These correlations link innate immune genetics and complement regulation to variations in clinical outcomes, but they are still indicators of susceptibility rather than proof of a direct causal mechanism.

Certain inflammatory indicators, platelet counts, complement measurements, and host genetic variations were linked to the severity or outcome of the disease in all of these cohorts. None have yet received independent validation as a prognostic marker and these correlations do not prove causation [35,36,37,38].

## 3. Transmission Biology

### 3.1. Reservoir Spillover

The ANDV natural rodent reservoir, the long-tailed pygmy rice rat (*O*. *longicaudatus*), which is found throughout southern Chile and Argentina, is strongly linked to the virus’s emergence [5,39]. However, the presence of infected rodents is not the only factor that affects human spillover. In regions with particular temperature and precipitation patterns as well as considerable rodent species richness, ecological niche analyses revealed significant overlap between seropositive rodents and human HCPS cases, indicating that these environmental factors may promote hantavirus spillover to humans [3]. Additional evidence for this ecological view comes from Chilean fieldwork. Ninety-two peridomestic and rural locations from Coquimbo to Aysén (30–45° S) were assessed in a 19-year survey of locations linked to human HCPS cases. Fifty-eight out of 1847 collected small mammals had anti-ANDV antibodies, with *O*. *longicaudatus* exhibiting the greatest seropositivity among the rodents in the sample [40]. *O*. *longicaudatus* uses a variety of habitats, including open, shrub-land, ecotonal, and wooded areas. In northwestern Chubut, Argentina, *O*. *longicaudatus* was found in steppe, ecotone, and forest areas, including sweet-briar shrublands, *Austrocedrus* forests, and *Nothofagus* forests. These results suggests that HCPS risk is not limited to closed forest environments, since ANDV antibodies were identified across all regions, albeit not in every landscape unit, and the researchers reported that human HCPS cases occurred not only in forests and shrublands but also in steppe [41]. Changes in peridomestic environment are especially important for human exposure. When rural peridomestic environments in northwestern Patagonia were compared to adjacent sylvan habitats, *O*. *longicaudatus* was the most prevalent rodent in peridomestic settings, accounting for at least half of the caught individuals [5]. The probability of finding an ANDV-positive rat was 2.44 times higher in peridomestic environments compared to sylvan habitats, indicating that altering rural ecology can promote the presence of reservoirs and raise the likelihood of human contact with infected rodents [5]. *Abrothrix longipilis*, *A*. *olivaceus*, and *Loxodontomys micropus* are among the other sympatric sigmodontine rodents that have also been connected to ecological variables associated with HCPS risk [3]. However, these relationships should not be taken as establishing them as equal primary reservoirs. Overall, the investigations suggest that *O*. *longicaudatus* is the major reservoir and that the most likely locations for zoonotic exposure are determined by reservoir abundance, habitat change, seasonality, and geography.

### 3.2. Viral Shedding and Infectiousness

Transmission potential after spillover is dependent on whether infected hosts release the virus through compartments that can infect another susceptible host. Tissue research offers biological evidence for salivary and respiratory pathways. A study looked at both fatal human HCPS cases and spontaneously infected animals and showed that ANDV antigen was widely distributed in lung macrophages and alveolar epithelial cells [41]. The biological plausibility of respiratory and salivary shedding routes was further supported by the discovery of viral antigens in secretory cells and luminal contents of the submandibular salivary glands [41]. A study’s findings challenged the conventional belief that urine is the main source of transmission by detecting ANDV immunoreactivity in rodent lungs, primarily in alveolar epithelial cells and macrophages. They also found strong immunoreactivity in salivary glands with relatively limited involvement of the urinary tract [42]. Further evidence of horizontal transmission between Syrian hamsters after close contact was provided by another study which showed that contact animals developed productive infection and detectable ANDV RNA in organs [43]. Viral RNA identification and/or seroconversion demonstrated that all hamsters exposed to ARG-Epuyén in direct-contact experiments contracted the infection [44]. This shows effective transmission in the laboratory setting but does not prove higher transmission fitness in human populations. While the Epuyén outbreak offers epidemiological evidence of persistent human-to-human transmission, comparable epidemiological or virological evidence would be needed to attribute this trend to an inherent advantage of the strain [45].

Saliva, gingival crevicular fluid, nasopharyngeal swabs, plasma, and buffy coat have all been found to contain ANDV RNA during acute infection; positivity is more common early in the illness, indicating potential transitory shedding from oral or respiratory locations [45]. Isolation of a replication-competent virus from an early buffy coat sample collected prior to the development of detectable anti-ANDV antibodies provides stronger virological evidence [46]. Additionally, ANDV RNA has been found in semen following recovery; however, this data does not prove sexual transmission or live virus [47] (Table 1).

### 3.3. Host Immunity and Transmission Potential

The evidence examined here can be divided into three groups. First, the aforementioned experimental studies reveal that ANDV can disrupt innate antiviral signaling [8,17,18,19,20,21,22,23]. These results establish mechanisms in laboratory systems, but they do not prove that human infectiousness is determined by interferon antagonism. Second, clinical research links the development of immunological responses, such as activated T-cell and neutralizing-antibody responses, to delayed viral clearance [27]. Virological evidence of a potentially infectious phase is provided by the recovery of a replication-competent virus prior to detectable antibodies [46]. These results relate to viral kinetics and immunological responses; they do not demonstrate that any virological or immune signal predicts further transmission.

Third, epidemiological research shows that human-to-human transmission varies. Secondary transmission was extremely overdispersed during the Epuyén outbreak, with a disproportionate number of subsequent cases being caused by a limited number of symptomatic persons [48]. Even though this clustering shows variations in infectiousness among people, the current epidemiological data cannot differentiate the impacts of host immunity from viral load, viral strain, exposure timing, contact duration, or contact intensity. Furthermore, despite reduced replication in vitro, an Epuyén-associated strain remained effectively transmissible, suggesting that replication rate or any one host reaction alone is unlikely to account for transmission fitness [12].

Therefore, a cautious interpretation of the current evidence is warranted. While growing adaptive immunity may aid in viral clearance, delayed antiviral control may theoretically increase the amount of time a viable virus is available for transmission; both suggested connections with the length of human infectiousness are still speculative. No direct connection between immunological reactions and transmission has been established. No immunological signature has yet been demonstrated to differentiate people who spread ANDV from those who do not when the timing and severity of exposure are taken into account.

## 4. Epidemiological Evidence for Human-to-Human Transmission

### 4.1. Early Evidence from Argentina and Chile

Epidemiologically linked clusters in Chile and Argentina provided early proof of ANDV human-to-human transmission. These findings should be interpreted cautiously since the strength of transmission inference varies per cluster and depends on factors such as the consistency of exposure timing, the level of close contact, viral sequence relatedness, and the possibility that shared rodent or environmental exposure was eliminated. A pioneering study examined four Argentine clusters and showed that close contact with symptomatic patients led to the development of secondary HCPS cases in Cluster 1 (C1) and Cluster 4 (C4). 100% viral nucleotide identity was observed among linked subjects, strongly suggesting interhuman transmission rather than distinct rodent exposures [49]. This evidence was reinforced by prospective household-contact research in Chile with 476 household contacts of confirmed HCPS patients. In contrast to other home contacts, sex partners had a much-increased chance of developing HCPS, indicating that close-contact transmission rather than casual household exposure is more likely to result in transmission [50]. In a similar vein, another study examined 215 family and healthcare contacts of Chilean HCPS patients and discovered seropositivity in just 1.9% of family contacts and none among healthcare workers, indicating that ANDV transmission can occur but comparatively ineffective and heavily reliant on close exposure [51]. A Chilean cluster was later reported in which close contact with symptomatic patients led to the infection of a spouse, two healthcare workers, and a secondary home contact, supporting person-to-person transmission [8].

### 4.2. Epuyén Outbreak

The 2018–2019 Epuyén outbreak in Argentina’s Chubut Province offers one of the most thorough reconstructions of ANDV transmission from person to person. It is more insightful to interpret the outbreak as a heterogeneous transmission network rather than as a series of social activities. The outbreak, which comprised 34 confirmed infections and 11 fatalities, was compatible with a single zoonotic introduction followed by four reconstructed waves of human transmission [45]. Transmission was significantly clustered, with three symptomatic persons accounting for twenty-one out of thirty-three secondary cases, suggesting that onwards spread was not evenly dispersed throughout the community but rather localized around high-contact exposures [45]. This perspective is also supported by epidemiological parameters. Fifty-two percent of secondary transmission occurrences were associated with exposure on the first day of fever in the inferred source case, with a mean serial interval of roughly 23 ± 7 days and incubation durations ranging from 9 to 40 days [45]. Prior to control measures, reproductive-number modeling showed a reduction from 2.12 to 0.96 following the isolation of confirmed cases and contacts’ self-quarantine. The decline may have been driven by other concurrent events; therefore, this temporal connection does not prove that these measurements alone were responsible [45]. Individual transmission links should still be viewed cautiously though, as several cases had multiple potential sources and reconstruction relied on established and likely epidemiological associations. All things considered, Epuyén demonstrates that ANDV person-to-person transmission is diverse, dependent on close contact, and significantly influenced by public health intervention (Table 2).

### 4.3. Genomic Epidemiology

Genomic epidemiology is becoming a crucial tool for differentiating between shared environmental exposure and direct person-to-person transmission. Nevertheless, genomic similarity should be taken as supporting evidence of relatedness rather than independent proof of direct transmission, particularly in endemic regions where closely related viruses may circulate in local rodent populations. A study used whole-genome sequencing to examine a three-case Argentine cluster from 2014 and found 100% nucleotide identity amongst epidemiologically related cases, including an illness connected to hospital exposure. 99.95% identity in the L segment and full identity in the S and M segments were displayed in the remaining case. This study was particularly significant because previous studies were mostly based on partial viral sequencing [7]. The Epuyén out-break later yielded more genetic evidence. Using phylogenetic analysis and next-generation sequencing, a study showed that 78% of sequenced cases had the same core viral haplotype, with viral sequences exhibiting 99.8–100.0% similarity during the Epuyén outbreak. These results were consistent with a single spillover occurrence and subsequent person-to-person transmission [44]. More recently, utilizing whole-genome sequencing, a study discovered a novel Chilean ANDV haplotype from an asymptomatic close family contact during early infection [45]. In a similar vein, another study examined ANDV sequences from rodents and HCPS patients throughout Chile and discovered two significant viral clades associated with various Chilean ecoregions by examination of the S and M genome segments [4].

Despite the growing evidence of ANDV human-to-human transmission, a systematic review discovered that the majority of the research that were accessible were epidemic investigations and noncomparative cluster analyses, with just a small number using comparative designs. Exposure misclassification and the challenge of completely ruling out shared rodent exposure in a number of documented transmission clusters were among the significant shortcomings that were also brought to light [53]. All things considered, the information that is now available should be taken as showing that ANDV can spread between individuals, but that this spread is rare, comparatively ineffective, and concentrated in intimate interpersonal exposure during early symptomatic illness.

## 5. High-Risk Exposure Settings

### 5.1. Household and Intimate Exposure

Household-contact evidence suggests that frequent cohabitation is not as closely associated with ANDV transmission within homes as intensive and persistent interpersonal encounters. Spouses, sexual partners, and caregivers were consistently found to be the highest-risk contacts in epidemiologic studies from Chile and Argentina, especially when exposure involved sharing sleeping areas, extended indoor contact, or direct care during early febrile illness. A study found that sexual partners had a much higher risk of HCPS than other home contacts [50]. This suggests that intimate or sustained close contact carries a higher risk of ANDV transmission than casual household contact.

### 5.2. Healthcare Exposure

Although healthcare-associated ANDV transmission is uncommon, a number of studies indicate that infection may happen when medical personnel without appropriate personal protective equipment having close, continuous contact with symptomatic patients prior to the clinical diagnosis of HCPS. One of the most compelling cases of nosocomial transmission was reported in a study in which two medical personnel contracted HCPS after tending to an infected patient [8]. A genomically linked Argentine cluster that includes a case related to healthcare after hospital exposure was subsequently described in another study [7]. These reports suggest that direct patient care may increase the risk of transmission in clinical settings compared to regular hospital inter-action alone. Close contact with seriously unwell individuals represents the highest-risk scenario, especially in the early stages of febrile infection when identification and isolation may be postponed. During supportive treatment, exposure to contaminated clinical items, oral fluids, or respiratory secretions may significantly raise the risk. All of the information that is currently available suggests that healthcare-associated ANDV transmission can occur, but it is rare and seems to necessitate direct, prolonged, or high-exposure contact with symptomatic patients or their bodily fluids.

### 5.3. Confined Travel Environments

Confined travel environments may raise the risk of ANDV transmission not only because they open up new routes of transmission but rather because they exacerbate factors associated with person-to-person transmission, such as extended close contact, shared cabins and airspace, delayed detection of early symptoms, and challenges in separating exposed travelers. Delayed detection may occur because early ANDV symptoms are non-specific and may mirror typical travel-related viral disease, while a rodent-borne hantavirus infection may not be immediately anticipated in a cruise context. Instead of a standard onboard diagnosis, confirmation may also necessitate laboratory coordination and public health notification [52]. Passengers and crew frequently spend long stretches of time together in enclosed social areas during cruise and expedition trips, which increases the likelihood of exposure before illness is detected. This risk in a contemporary travel environment is highlighted by the MV Hondius ANDV cluster.

According to the Andes Virus Outbreak Working Group (2026) [54], an early severe case experienced fever and dyspnea on 21 April and was medically evacuated from the MV Hondius to Ascension Island on 27 April due to a severe acute respiratory infection. The case showed evidence of pneumonia, even though initial chest radiography was unremarkable. When his condition worsened, he was sent to Johannesburg for ventilator support and critical care, where the results were consistent with atypical pneumonia [52]. The results of the diagnostic evaluation, which included blood cultures, fungal biomarkers, malaria smear and antigen, respiratory pathogen panels, and legionella urine antigen, were inconclusive. This case demonstrates why ANDV may not be suspected early in cruise settings since prodromal and early respiratory symptoms often match more prevalent travel-related diseases [52].

The ensuing multinational investigation made the public-health implications of this constrained travel-related incident more evident. Three deaths and a 23% case-fatality rate were found among the thirteen out-break-associated patients, which included one possible infection and twelve laboratory-confirmed infections. Passengers (10/121) and crew personnel (3/61) were involved in cases, and people of 23 different nationalities required medical evacuation, repatriation, coordinated international contact tracing, isolation, quarantine, and laboratory and clinical follow-up [9]. A geographically limited outbreak might result in a significant global surveillance burden after travelers disperse, as demonstrated by the tracing of over 600 contacts over 32 countries and territories [55].

Crucially, neither ventilation nor shared indoor spaces were established as separate trans-mission pathways by the inquiry. The most plausible explanation for the outbreak was an initial zoonotic illness that occurred before embarkation, followed by secondary and maybe tertiary person-to-person transmission on board. Twelve laboratory-confirmed cases and one suspected case were among the 13 outbreak-associated cases that have been recorded as of 2 July 2026. No new secondary cases were found, and all known contacts had finished the follow-up. WHO deemed the outbreak contained, despite the fact that the exact pathways of transmission and the original source of infection were still unknown.

## 6. Public Health Implications

### 6.1. Early Recognition and Diagnosis

In order to prevent rapid cardiopulmonary deterioration, early detection should concentrate on identifying ANDV infection during the non-specific prodromal phase. The majority of patients first exhibit symptoms that resemble common viral or gastrointestinal disorders, such as fever, myalgia, headache, exhaustion, abdominal discomfort, nausea, vomiting, or diarrhea. However, a number of clinical trials from Argentina and Chile revealed that severe HCPS is often preceded by early thrombocytopenia, leukocytosis, raised hematocrit, rising lactate, and new hypoxemia [56]. Many individuals rapidly deteriorated after this early febrile phase [57], while other research showed that a lower platelet count at admission could potentially determine individuals at risk of progressing from initially mild ANDV disease to more severe HCPS [54]. Because exposure history frequently offers the first indication of clinical concern, it is equally significant. Clinicians should enquire about recent travel to southern Chile or Argentina, stays in rural cabins, work in forestry or agriculture, rodent infestation, cleaning of confined spaces, and recent close contact with a person who has a fever or respiratory symptoms. The diagnostic difficulties of ANDV infection in returning travelers were brought to light by im-ported European cases, since early symptoms were non-specific and the diagnosis mostly depended on identifying pertinent travel and exposure history [58]. Probable ANDV cases in healthcare settings segregated as soon as an ANDV infection is suspected, treated with conventional and respiratory precautions, and tested as soon as possible using RT-PCR or serology, if available.

### 6.2. Isolation and Infection Control

Recommendations from published research highlighted that healthcare precautions for suspected ANDV infection should start as soon as the infection is clinically detected since early symptoms are non-specific and respiratory deterioration can happen quickly. It is still crucial to take standard precautions including washing your hands, sanitizing your surroundings, handling blood and bodily fluids safely, and wearing the proper PPE. According to CDC’s 14 May 2026 interim guidance, patients with known or suspected ANDV infection should be placed in an airborne infection isolation room (AIIR). When entering the patient’s room, they should wear a gown, gloves, eye protection, and an N95 or higher-level respirator [59]. Patients should only be moved when transportation is medically necessary; in such cases, the patient should wear a mask, and personnel should utilize personal protective equipment (PPE) throughout the transfer.

### 6.3. Tracing and Monitoring

**Figure 3:**
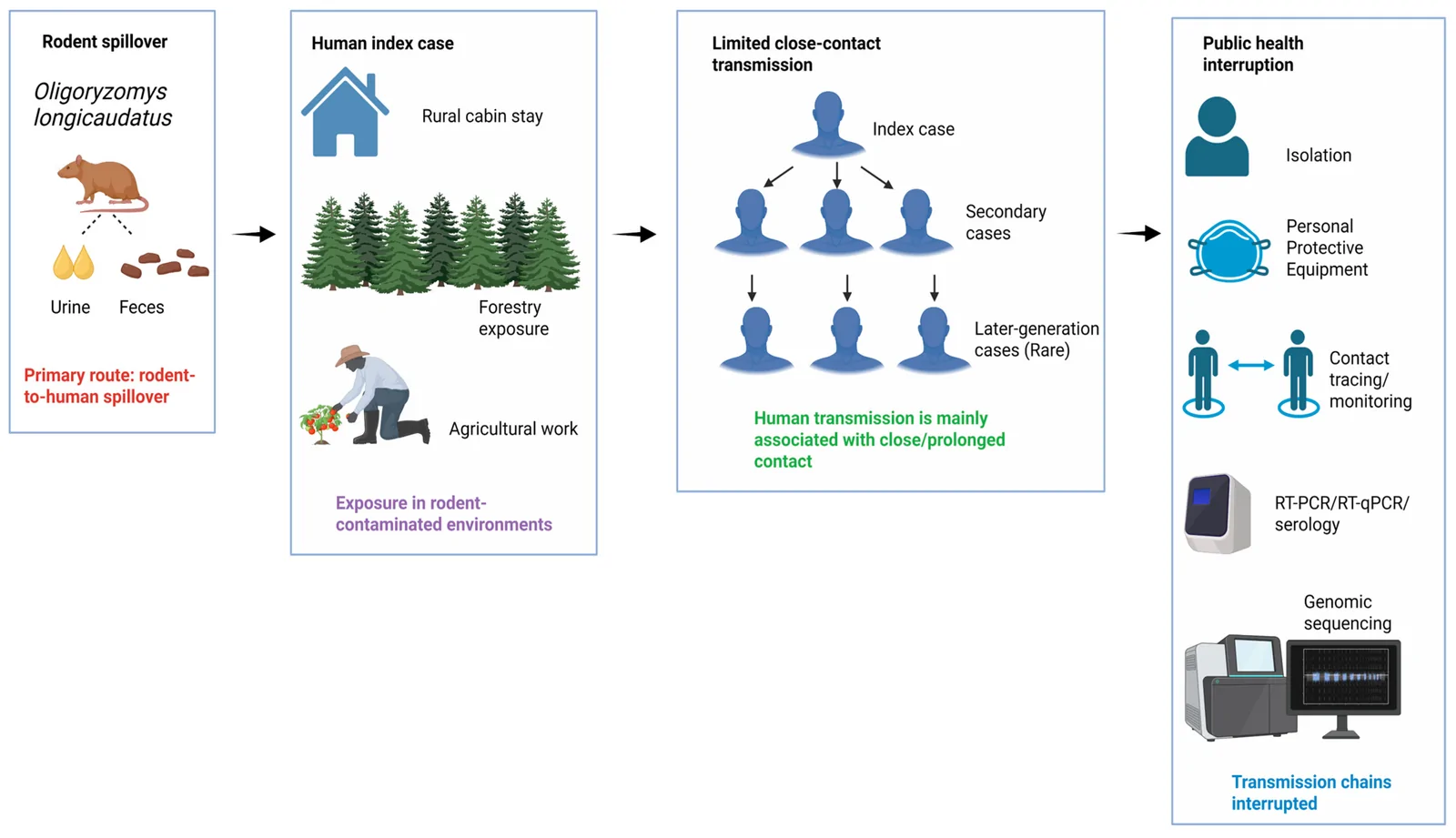
Andes virus transmission and outbreak control overview. Created in BioRender. Ibrahim, M. (2026) https://BioRender.com/a74em59 (accessed on 27 July 2026).

ANDV contact tracing ought to be risk-stratified based on the degree of exposure. While lower-risk contacts can be mostly handled with symptom monitoring, high-risk contacts, such as household members, caretakers, and those with extended near or indoor exposure, may require quarantine and serial RT-PCR [53]. The extended incubation period supports monitoring for up to 42 days following the last pertinent exposure. Additionally, the MV Hondius outbreak demonstrated that ANDV RNA can be found before the development of symptoms; however, it is still unclear if presymptomatic RNA discovery is indicative of infectiousness or subsequent transmission [55]. These results are in favor of long-term, exposure-based surveillance as opposed to consistent monitoring of every interaction, especially when exposed persons spread throughout several jurisdictions (Figure 3).

### 6.4. Cross-Border Response

Exposure during international travel could quickly turn a limited outbreak into a cross-border coordination difficulty, as the 2026 MV Hondius ANDV cluster showed. People from 23 different countries were affected by the outbreak, which necessitated coordinated medical evacuation, repatriation, international contact tracing, isolation, quarantine, and clinical and laboratory follow-up across several jurisdictions. After passengers and crew had dispersed, laboratory-confirmed illnesses were found among travelers assessed in various nations, necessitating the quick interchange of epidemiological and diagnostic data across national public health agencies [9].

Repatriation constituted a significant operational component of the response with further isolation, quarantine, and follow-up ongoing within destination nations [9]. A comparatively small number of travel-associated ANDV cases can result in a significant worldwide monitoring burden, as evidenced by the more than 600 contacts that were ultimately identified across 32 countries and territories [55]. All of these findings point to the necessity of prompt case and exposure information sharing, coordinated management of potentially exposed travelers, and ongoing contact tracing and laboratory surveillance after international dispersal for the successful cross-border management of travel-associated ANDV events [9,55].

## 7. Future Research Priorities

Prospective studies that address present transmission attribution constraints should be given priority in future ANDV research. Incomplete contact ascertainment, uncertain zoonotic exposure, inconsistent timing of virological sampling, and limited serial sampling were found to be significant methodological flaws in a systematic review of 33 studies that included 17,204 individuals, 2221 laboratory-confirmed cases, and 135 secondary cases. Therefore, standardized exposure assessment should be combined with serial virological sampling and genomic analysis in future contact studies. To better differentiate person-to-person transmission from shared zoonotic exposure, viral sequence similarity should be read in conjunction with clearly characterized epidemiological and temporal linkages [60].

Additionally, prospective studies must validate immune correlates. Higher Gn-specific IgG levels, stronger antibody-dependent NK-cell activation, and complement deposition were linked to intermediate disease compared to severe disease in 14 Chilean patients with acute HCPS. Years after infection, polyfunctional antibody activity was still present in 34 survivors [34]. Instead of just reflecting variations in clinical outcome, these putative markers should be assessed at standardized early time points in larger independent cohorts to see if they might predict disease progression.

There is still little clinical data. Persistent gaps in validated point-of-care diagnosis, disease-specific treatment, and long-term outcome data were found in a 2026 scoping assessment of 40 papers, most of which were retrospective cohorts and case reports from Chile and Argentina. Therefore, defined disease-stage criteria and common clinical objectives should be used in prospective multicenter trials to assess diagnostic performance and therapy results [61].

Research on prevention ought to go beyond early-phase immunogenicity. The ANDV DNA vaccine was safe and produced long-lasting neutralizing responses in 48 healthy adults between the ages of 18 and 49, according to a randomized phase 1 experiment; nevertheless, conclusions regarding its effectiveness are limited by the small treatment groups and lack of a proven human correlate of protection [62].

Lastly, the low-passage ARG-Epuyén isolate from a human case in the 2018 outbreak offers an epidemiologically important platform for developing countermeasures. More characterization is needed before it may be considered a wild-type challenge strain for testing potential vaccines and treatments [44].

## 8. Conclusions

The degree of ANDV illness and person-to-person transmission are linked but distinct outcomes. Host immune responses have been linked to illness severity and viral control while transmission studies show diverse secondary spread and the identification of viral RNA or, less frequently, replication-competent virus. However, there is currently insufficient data to conclude that immunological responses linked to severe illness cause, extend, or predict infectiousness. Whether these reactions affect the duration or timing of the infectious phase and, consequently, the probability of secondary transmission is the main unanswered question. Prospective studies combining exposure assessment, genomic analysis, and serial immunological and virological assessments are required to differentiate between factors that define infectiousness and markers of illness severity.

## Figures and Tables

**Table 1:** Evidence of Andes virus detection and infectivity in biological specimens and experimental models.

Specimen or Model	Detection Method	Study Population	Principal Finding	Interpretive Limitation	References
Salivary-gland tissue	Immunocytochemistry, immunofluorescence, and transmission electron microscopy	Fatal human HPS cases and ANDV-infected rodents	Submandibular salivary gland luminal contents and secretory cells were found to contain ANDV antigen; these results were in line with infection and replication in these tissues.	The presence of antigen in gland tissue does not indicate the presence of an infectious virus in saliva or prove salivary transmission.	[41]
Saliva	RT-qPCR; cell-culture infectivity assay of RT-qPCR-positive fluids	Prospective cohort of 131 people with confirmed ANDV infection in Chile, enrolled 2008–2022	Saliva from both acute and convalescent samples contained ANDV RNA. A fraction of positive bodily fluid samples, including saliva, tested positive for infectivity.	RNA detection by itself does not demonstrate viability. The infectivity of the examined sample is supported by a positive culture assay; however, this does not prove that saliva spreads virus between individuals.	[48]
Nasopharyngeal swabs	RT-qPCR; cell-culture infectivity assay of RT-qPCR-positive fluids	Same prospective Chilean cohort	16% of acute phase samples and 2% of convalescent phase samples had ANDV RNA. In certain nasopharyngeal samples that were RT-qPCR positive, infectivity tests also revealed the presence of an infectious virus.	Viability cannot be established by RNA detection alone. Although recovery in cell culture confirms the tested sample’s infectivity, it does not prove the path, frequency, or occurrence of human-to-human transmission.	[48]
Plasma	RT-qPCR	Same prospective Chilean cohort	Thirty percent of convalescent-phase samples and fifty-four percent of acute-phase samples had ANDV RNA in their plasma.	RNA testing confirms the presence of viral genetic material in plasma, but it does not prove that the virus was contagious.	[48]
Buffy coat	RT-qPCR; cell-culture isolation	Prospective Chilean cohort; separately, an asymptomatic household contact followed during a family-cluster investigation	In the prospective cohort, RNA was found in all acute-phase samples. In a different instance, replication-competent ANDV was isolated in Vero E6 cells from a day-1 buffy-coat sample that was obtained prior to the detection of anti-ANDV antibodies.	Viability cannot be proven by RNA positivity alone. A replication-competent virus is established in the tested sample through successful isolation, but transmission to another individual is not established.	[45,48]
Semen	RT-qPCR; attempted virus isolation	Longitudinal follow-up of one recovered male patient	ANDV RNA remained in semen for almost six years; attempts to isolate the infectious virus from the examined semen samples failed	RNA persistence does not prove sexual transmission or a live virus. Failure to isolate the systems under examination does not indicate the absence of infectious particles.	[46]
Syrian hamster contact model	Close-contact exposure followed by virological and serological testing	Experimentally exposed Syrian hamsters	Contact animals became infected and supported transmission under the experimental conditions	An animal model shows transmission within that model; it does not identify a human channel or measure the risk of human transmission.	[43]

**Table 2:** Evidence base for Andes virus human-to-human transmission.

Country/Year	Setting	Evidence Type	Main Finding	Reference
Argentina/2002	Four epidemiologically connected HCPS clusters across endemic areas	Epidemiologic reconstruction with G1/G2/S-NCR sequence comparison	Strong epidemiological and sequence-based evidence for interhuman transmission was shown by the C1 and C4 clusters.	[47]
Chile/2001–2005	Prospective household-contact cohort	Weekly clinical follow-up; serology; peripheral-blood RT-PCR	Intimate partners had the greatest risk of HCPS; ANDV RNA preceded symptoms and antibodies.	[49]
Chile/2011	Rural household and hospital-linked cluster	Epidemiologic investigation; IgM/RT-PCR confirmation; Gn/Gc sequencing	Epidemiologic and sequencing data supported four secondary cases, involving two nosocomial infections.	[7]
Argentina/2014	Three-case HCPS cluster	Target-enrichment whole-genome sequencing	P2–P3 transmission was supported by whole-genome identity, whereas P1–P2 transmission was still most likely.	[6]
Argentina/2018–2019	Epuyén outbreak with linked cases in Argentina and Chile	Contact tracing; RT-qPCR/serology; NGS; reproductive-number modeling	34 instances were generated by a single spillover, while 64% of secondary transmission was produced by three symptomatic patients.	[52]

Note: It is important to interpret each type of evidence independently. Epidemiological reconstruction suggests inferred exposure linkage; RT-PCR or RT-qPCR identifies viral RNA but does not establish an infectious virus or transmission pathway; infectious-virus isolation or infectivity evidence strengthens the case for a viable virus and genomic similarity suggests relatedness within a transmission network.

## Data Availability

No new data were created or analyzed in this study. Data sharing is not applicable to this article.
